# Clinical Manifestation of Subacute Thyroiditis Triggered by SARS-CoV-2 Infection Can Be HLA-Dependent

**DOI:** 10.3390/v13122447

**Published:** 2021-12-06

**Authors:** Magdalena Stasiak, Katarzyna Zawadzka-Starczewska, Andrzej Lewiński

**Affiliations:** 1 Department of Endocrinology and Metabolic Diseases, Polish Mother’s Memorial Hospital—Research Institute, 281/289 Rzgowska St., 93-338 Lodz, Poland; mstasiak33@gmail.com (M.S.); kasiula891@op.pl (K.Z.-S.); 2 Department of Endocrinology and Metabolic Diseases, Medical University of Lodz, 281/289 Rzgowska St., 93-338 Lodz, Poland

**Keywords:** subacute thyroiditis, SARS-CoV-2, COVID-19, HLA

## Abstract

In the last two years, we have been struggling with the pandemic of SARS-CoV-2, the virus causing COVID-19. Several cases of subacute thyroiditis (SAT) have already been described as directly related to SARS-CoV-2 infection. The clinical course of SAT induced by SARS-CoV-2 can be entirely different from the classic SAT course, and one of the most important differences is a very rapid SAT onset observed in some patients, especially a phenomenon of the simultaneous presence of both diseases. The aim of this report is to compare HLA profile and clinical course of SAT in four patients, in whom SAT was considered as triggered by COVID-19, with special attention paid to the differences between a patient with rare simultaneous presence of SAT and COVID-19, and patients with longer time lag between the diseases. The unusual phenomenon of simultaneous occurrence of COVID-19 and SAT induced by SARS-CoV-2 infection can be HLA-dependent and related to the presence of homozygosity at HLA-B*35. Additionally, the clinical course of SAT triggered by COVID-19 can be HLA-related in regard to the risk of recurrence, and to a variety of other aspects, including severity of thyrotoxicosis.

## 1. Introduction

Subacute thyroiditis (SAT) (also called granulomatous giant cell thyroiditis or de Quervain’s disease) is a destructive inflammatory thyroid disease triggered mainly by viral factors in genetically predisposed individuals [1,2]. 

One of the characteristic SAT symptoms is an anterior neck pain radiating to the mandible, ear and upper chest [1]. However, a painless course has been more and more often described [3,4]. Fever is common, not rarely exceeding 39 °C. Patients usually complain of fatigue and may also have symptoms of thyrotoxicosis. Laboratory tests show a characteristically accelerated erythrocyte sedimentation rate (ESR), usually together with an increased concentration of C-reactive protein (CRP) in the serum and laboratory signs of thyrotoxicosis. Ultrasound (US) reveals hypoechogenic areas with blurred margins and reduced vascularization [1,4].

Recently, it has been demonstrated that the risk of SAT development is associated not only with the previously reported HLA-B*35, but also with the presence of HLA-B*18:01, -DRB1*01 and -C*04:01 [2]. SAT can be triggered by several viruses, including Coxsackie viruses, Echo viruses, influenza viruses, adenoviruses, parvovirus B19, mumps and rubella viruses, orthomyxovirus, HIV, Epstein–Barr virus, hepatitis E, measles and dengue viruses [4,5,6]. Since winter 2019, SARS-CoV-2 pandemic has spread around the whole world, making COVID-19 the most urgently analyzed new infectious disease ever. Prior to the current pandemic, no correlation between coronaviruses and SAT development was reported [5]. SARS-CoV-2 was demonstrated to have significant tissue tropism, with a particularly high affinity to the thyroid tissue. The SARS-CoV-2 genome has been detected in 36% of autopsy thyroid tissue specimens from patients who died of COVID-19 [7]. This affinity results from the fact that thyroid cells are rich in an angiotensin converting enzyme 2 (ACE2), which serves as a “receptor” for SARS-CoV-2 [8,9,10]. SAT can probably result from both direct thyroid cell destruction and immune mediated damage caused by cytokines and other inflammatory factors [11]. Since the beginning of the COVID-19 pandemic, many cases of SAT induced by COVID-19 have been described [4,12,13,14,15,16,17,18,19,20,21,22,23], and the virus has been included in the list of viral SAT triggers [24]. After two years since the pandemic onset, it has become clearly visible that the clinical course of SAT induced by SARS-CoV-2 can be entirely different from the classic one. Painless course occurs more often, while tachyarrhythmia and exacerbation of a general condition are quite frequently described as main manifestations, especially in patients hospitalized due to COVID-19 [4,12,15]. SAT occurs after approximately 2–6 weeks from either symptomatic or asymptomatic viral infection. In SARS-CoV-2 infection, there is a greater variability: in some cases, few days may elapse from the onset of COVID-19 to the development of SAT [16], and both diseases may occur simultaneously [4,12,15]. Additionally, in such cases, SAT may sometimes be the only symptom of active SARS-CoV-2 infection [17,18]. On the other hand, in some patients, SAT begins typically several weeks after COVID-19 [4,19]. No data are currently available on the potential key factors which may influence the phenomenon of such a different time lag between the SARS-CoV-2 infection and SAT. 

The aim of this study is to compare the HLA profile and clinical course of SAT in four patients in whom SAT was considered as triggered by COVID-19. Special attention has been paid to the differences between a patient with the rare simultaneous presence of SAT and COVID-19, and patients with a longer time lag between the diseases. 

## 2. Case Presentation

### 2.1. Case 1

From 6 October 2020, a 50-year-old man experienced neck pain (more intense on the right side, with a palpable hard neck tumor), fever and headaches. Treatment with 500 mg of azithromycin daily was introduced by a general practitioner (GP), but no symptom relief was observed. Additionally, after a few days, the patient reported a non-productive cough and the fever increased up to over 39 °C. The treatment was changed to 2.0 g of amoxicillin daily. A day later the patient noticed a significant malaise, loss of smell and retrosternal discomfort. He was admitted to the emergency unit where an active SARS-CoV-2 infection was confirmed by a PCR test. Cardiac tests did not reveal any abnormalities. At discharge from the emergency unit, azithromycin, 250 mg/day was recommended. There was no improvement from the treatment, and the dose of azithromycin was increased to 500 mg/day by the GP. Instead of symptom relief, the patient reported worsening of fever, cough, headache, insomnia and neck pain, with palpable hard neck tumor. Blood laboratory tests were not performed at that time, but due to the lack of efficacy of the previous therapies and the diagnosis of COVID-19, a treatment with dexamethasone 4.0 mg daily was introduced, with further gradual reduction of the dose. In the following days, the patient’s general condition improved significantly. About 4 days after the end of the steroid therapy, the symptoms reappeared, with a palpable hard tumor on the right side of the neck, insomnia and headaches, tachycardia up to 120 beats per minute and an increased blood pressure even to 170/115 mm Hg. Due to the re-occurrence of fever, the GP recommended treatment with cefuroxime 1000 mg/day. Finally, after over a month of nearly constant presence of neck pain, palpable hard neck tumor and fever, a neck ultrasound (US) was performed and revealed a hypoechoic, heterogeneous 20 mm × 18 mm × 16 mm focal lesion of the right thyroid lobe, without decreased vascularity in the color Doppler examination. On the basis of laboratory tests (Table 1), US pattern and fine needle aspiration biopsy (FNAB) results, the patient was finally diagnosed with SAT and a treatment with prednisone was started, with the rapid reduction of symptoms and gradual normalization of inflammatory parameters. After gradual prednisone dose reduction and further treatment withdrawal, no symptom recurrence was observed for 10 months of follow-up. The patient’s HLA profile revealed very strong genetic susceptibility to SAT (Table 2), with very rarely observed homozygosity at HLA-B*35.

### 2.2. Case 2

A 39-year-old woman was diagnosed with COVID-19 in December 2020. The disease course was of moderate severity, with typical symptoms of cough, headache, fever and malaise. Five weeks after the positive result of the PCR test, a fever and malaise occurred again, together with severe neck pain, tachycardia, insomnia and tremor. Laboratory tests revealed increased inflammatory markers and severe thyrotoxicosis without increased levels of anti-thyroid antibodies (Table 1). Thyroid US pattern was typical of SAT. In the HLA typing, the genetic predisposition for SAT was found, and the patient had only one high risk allele, HLA-B*35:03 (heterozygote) (Table 2). Prednisone treatment was introduced at the starting dose of 60 mg daily with slight symptom relief. However, every attempt to reduce prednisone dose resulted in the exaggeration of symptoms and worsening of laboratory results. Thus, the dose was reduced very slowly under strict control of laboratory results and US image, with complete prednisone withdrawal after four months of treatment. No recurrence was observed for the 8 months of follow-up.

### 2.3. Case 3

A 55-year-old woman was diagnosed with SAT in November 2020 with typical clinical symptoms, laboratory results and FNAB confirmation. In the HLA typing, the genetic predisposition was found with the presence of two high risk alleles (Table 2). Two weeks after the beginning of prednisone treatment, she was infected with SARS-CoV-2 by her family member. The infection was confirmed by PCR test and COVID-19 was of moderate severity with typical clinical symptoms. Prednisone treatment was continued throughout the whole time of COVID-19 and further on, according to the initially recommended schedule. The patient completed prednisone therapy a month after the PCR positive test. At the moment of the therapy completion, her ESR was 4 mm/h and her thyroid US pattern was normal. The symptoms of SAT recurred after a week, with neck pain as the first manifestation. Due to the gradual worsening of symptoms, typical laboratory and US manifestation, the SAT recurrence was diagnosed and re-treatment with prednisolone was introduced. The clinical and laboratory characteristics of the patient is presented in Table 1.

### 2.4. Case 4 

A 57-year-old woman was diagnosed with COVID-19 in October 2020. The disease course was mild with common symptoms. Four months after the positive result of the PCR test, typical SAT symptoms, i.e., neck pain radiating to the ear, moderate fever, tachycardia and mood changes occurred. Laboratory tests showed highly increased inflammatory markers and thyrotoxicosis, and levels of anti-thyroid antibodies were normal (Table 1). Thyroid US pattern was typical for SAT. In the HLA typing, the genetic predisposition was found with the presence of three high risk alleles (Table 2). No homozygosity at these alleles was found. Prednisone treatment was introduced at the starting dose of 40 mg daily with very good response. After gradual dose reduction, the treatment was finished with complete resolution of clinical, laboratory and US signs and symptoms of SAT.

## 3. Material and Methods

### 3.1. SAT Diagnosis Procedures

The SAT diagnostic criteria were consistent with the ones recently proposed by our research team [4] and included: elevation of ESR (or at least CRP) plus hypoechoic area/areas with a blurred margin and decreased vascularization in US plus FNAB confirmation of SAT or at least FNAB exclusion of malignancy, plus at least one of the following: hard thyroid swelling and/or pain and tenderness of the thyroid gland/lobe and/or elevation of serum FT4 and suppression of TSH.

DNA was extracted from the peripheral blood. *HLA-A*, -*B*, -*C*, -*DQB1* and -*DRB1* were genotyped with the application of a next-generation sequencing method on Illumina platform (Illumina, San Diego, CA, USA). Sequencing-based HLA typing of all the HLA genes was carried out in a 96-well format within a semi-automated workflow by using MiaFora Flex5 typing kits (Immucor, Peachtree Konars, GA, USA). The sequencing results were analyzed by MiaFora NGS software. Data were considered sufficient for analysis whenever the coverage reached 40 and the number of cReads exceeded 50,000.

Serum concentrations of TSH and FT4 were measured by electrochemiluminescence immunoassay (ECLIA), Cobas e601 analyzer (Roche Diagnostics, Indianapolis, IN, USA). ESR was determined with Ves-Matic Cube 30 (Diesse, Monteriggioni, Tuscany, Italy), CRP was analyzed by VITROS^®^4600 Chemistry System (Ortho Clinical Diagnostics, Raritan, NJ, USA).

Ultrasound imaging was performed using a 7–14 MHz linear transducer (Toshiba Aplio XG; Toshiba, Japan). In all the cases, FNAB was performed using a 23-gauge needle. The presence of multinucleated giant cells together with mononucleated macrophages, and follicular epithelial cells with acute and chronic inflammatory dirty background (cellular debris and mixed inflammatory cells) was considered a cytology result typical of SAT.

### 3.2. Consent Procedures

The patients gave informed written consents for all the procedures performed and a signed consent to the publication for their medical data. The consent form was accepted by the Bioethics Committee of the Polish Mother’s Memorial Hospital—Research Institute, Lodz, Poland (approval code 108/2018).

## 4. Discussion

Since the beginning of COVID-19 pandemic, many case reports on SAT triggered by SARS-CoV-2 infection have been published [12,13,14,15,16,17,18,19,20,21,22,23]. The clinical course of SAT caused by COVID-19 can be different from the typical one [4], especially with regard to the time lag between the SARS-CoV-2 infection and SAT onset. Before the COVID-19 era, SAT had been believed to occur 2–6 weeks after viral infection. In SARS-CoV-2 infection, SAT may appear so rapidly after the infection that the clinical symptoms of both diseases may overlap, or SAT symptoms may even be the first—and sometimes the only—manifestation of the infection [4]. Such a phenomenon has never been observed before in regard to any virus which can potentially induce SAT. However, in many patients, SAT still begins typically a few weeks after COVID-19. Until now, no potential factors influencing this phenomenon have been proposed.

In our previous reports, we demonstrated that the type of genetic susceptibility related to HLA haplotype can be significantly associated with SAT course. Such correlation has been described in regard to US pattern of SAT [25] and to the risk of recurrence [26]. We observed that the specific type of HLA profile influences the course of SAT [25,26,27]. The coexistence of HLA-B*35 and -B*18:01 was demonstrated to be a marker of a high risk of SAT recurrence [26], while the presence of HLA-B*18:01 without any other SAT susceptibility alleles was associated with atypical US pattern [25].

The present report demonstrates the first observation in the literature that the atypically short time lag between SARS-CoV-2 infection and SAT onset may also be related to HLA. In Patient 1, SAT symptoms of neck pain with palpable hard thyroid swelling (the patient’s observation) and fever preceded COVID-19 clinical manifestation, which fully appeared a few days later (non-productive cough, loss of smell). At the time of COVID-19 symptom onset, the SARS-CoV-2 infection was confirmed by PCR test. In this patient, the genetic susceptibility for SAT was extremely strong (Table 2), with alleles belonging to two independent classes of the major histocompatibility complex (MHC). However, the key factor that is specific for this case and can possibly underlie such an early development of symptoms is the homozygosity at HLA-B*35 (Table 2). Homozygosity at SAT high-risk alleles is very rarely observed. In our cohort of SAT patients, the frequency of homozygosity at HLA-B*35 was approximately 3%, while the presence of HLA-B*35 heterozygosity was nearly 70% (unpublished data).

The hypothesis of homozygosity as a key factor can be supported by the HLA patterns of the three other presented cases. In Patient 4, the genetic susceptibility for SAT was proved, but the time lag between COVID-19 and SAT onset seemed to be too long to confirm the potential correlation between the diseases. However, as the SARS-CoV-2 infection can lead to the so called post-acute sequalae of SARS-CoV-2 infection (PASC) [28], the correlation cannot be unequivocally excluded. PASC is a syndrome in COVID-19 survivors that presents with a variety of symptoms in different organs. Long-term complications are not unexpected, taking into account the broad organotropism of SARS-CoV-2 and severity of tissue injuries [10]. PASC is a recently described COVID-19 complication, and a potential correlation between PASC and SAT has never been analyzed. The speculation of a possible prolonged time lag due to PASC requires further observation. However, regardless of whether SAT was triggered by some sequelae of distant consequences of SARS-CoV-2 infection, or, more likely, was not correlated with COVID-19 (i.e., induced by other factor), Patient 4 is a good candidate to compare with Patient 1 because her HLA profile was the same as the one of Patient 1, except for the homozygosity at HLA-B*35. Thus, this rare HLA-B*35 homozygosity seems to be the main possible candidate factor related to this rapid SAT onset, simultaneous with an acute phase of COVID-19. Another difference between these patients is gender, however, this factor can hardly be of any significance in regard to a time lag between COVID-19 and SAT onset [16,17,18]. A lack of thyrotoxicosis and only a moderate elevation of inflammatory markers in Patient 1 (Table 1) probably resulted from the previous application of dexamethasone. Despite the obvious SAT symptoms which preceded symptoms of COVID-19, the first diagnostic and therapeutic decisions were focused on infection treatment. Thus, antibiotics and anti-inflammatory agents, including steroids, were introduced before the first analysis of thyroid hormones and ESR. Therefore, laboratory results cannot be taken into account in the analysis of risk factors of rapid SAT onset in this patient. However, no specific difference in laboratory SAT markers was observed in patients with simultaneous SAT and COVID-19 presented in the literature so far [12,15].

Additionally, to further confirm a potential role of HLA haplotype in the phenomenon of rapid SAT onset in some of the COVID-19 affected patients, it would be valuable to compare HLA haplotype of Patient 1 with the haplotype of Patient 2, in whom SAT triggered by COVID-19 occurred typically 4 weeks after a positive PCR swab result. In this patient, in whom a direct correlation between SAT and COVID-19 was very highly probable, HLA-B*35:03 was present as the only SAT high risk allele. The time lag between COVID-19 and SAT was typical and consistent with previously described SAT triggered by viruses other than SARS-CoV-2 [4,5,29]. Additionally, in Patient 2, a severe laboratory and clinical thyrotoxicosis during the course of SAT could be also HLA related, but it seems to have no association with the time lag between COVID-19 and SAT. We have recently presented a report of SAT in three siblings with the coexistence of HLA-related predisposition for SAT and Graves’ disease (GD) [27]. However, GD occurred only in one of them, while the other two had a much more severe thyrotoxicosis in the course of SAT. Those two had a different HLA profile, and one of the differences was a lack of HLA-A*01:01 and -B*41:01. In Patient 2, similarly to these siblings, a predisposition to GD depended on the presence of HLA-DRB1*03:01. Moreover, the lack of HLA-A*01:01 and -B*41:01 together with the presence of severe thyrotoxicosis highly resembles those previously described cases and enables further confirmation of the crucial role of HLA pattern in the clinical course of SAT.

Patient 3 had SAT recurrence potentially triggered by COVID-19. The recurrence occurred despite prednisone treatment, which was started 2 weeks before SARS-CoV-2 infection and continued throughout the whole time of COVID-19 and further on, as initially recommended. A rapid relapse of SAT symptoms after steroid discontinuation is usually associated with too early treatment cessation. In such cases, residual lesions characteristic of SAT are typically still present in US [26]. Thus, in our center, US thyroid examination and levels of inflammatory markers are routinely controlled before steroid treatment cessation. At the time of treatment withdrawal, Patient 3 had low ESR (4 mm/h) and her thyroid US pattern was normal, with no signs of SAT residual lesions. Despite this full clinical, biochemical and US remission, the relapse of symptoms began only 7 days after steroid withdrawal and the correlation between SAT recurrence and COVID-19 seems possible. In this case, the time lag between the diseases could have been even shorter if the prednisolone treatment had been finished earlier. On the other hand, the disease did not relapse earlier during low dose prednisone therapy, despite the specific HLA profile—coexistence of HLA-B*35:01 and -B*18:01 (Table 2). Such a profile of HLA alleles was demonstrated to increase recurrence rate by approximately nine times, as compared to other SAT high risk profiles [26]. Therefore, Patient 3 was genetically predisposed to SAT recurrence, so the disease relapse was probable at some time in her life. However, it occurred with direct correlation to COVID-19, rapidly after steroid withdrawal. Thus, in this case, COVID-19 may be considered as the triggering factor and the time lag between the diseases and heterozygosity at HLA-B*35 further supports our hypothesis of HLA significance. Moreover, this patient constitutes a further confirmation of our published findings that the coexistence of HLA-B*35:01 and -B*18:01 is associated with a high risk of recurrence.

On the basis of the of the above presented data, it can be initially presumed that the simultaneous occurrence of COVID-19 and SAT triggered by SARS-CoV-2 infection as well as the observed very short time lag (a few days) between the diseases [4,16] can be HLA-dependent. The homozygosity at HLA-B*35 seems to be a possible key factor that can underlie such an early development of SAT symptoms. This observation requires further confirmation with larger patient cohorts. However, such a confirmation can be difficult due to the rarity of the described co-presence or extremely short time lag between COVID-19 and SAT. So far, this phenomenon has been reported in only a few patients worldwide. Unfortunately, HLA typing has not been performed in any of the described patients except for Patient 1. Among the small cohort of our patients with HLA-B*35 homozygosity, all SAT cases except for the one described above were diagnosed before the COVID-19 pandemic and in all those cases the time lag between viral infection and SAT was typically 2–3 weeks. As the simultaneous presence of SAT and viral infection has been described exclusively for SARS-CoV-2, the future confirmation of the role of homozygosity could probably be based on few case reports only.

A potential phenomenon of PASC-related prolongation of time lag between SARS-CoV-2 infection and SAT should be taken into account. However, this is only an initial speculation which requires further confirmation in a larger group of patients.

The simultaneous presence of COVID-19 and SAT, especially in the cases in whom SAT is the first manifestation of COVID-19, constitutes a significant diagnostic challenge. In a patient with SARS-CoV-2 infection, it is easy to overlook SAT symptoms, as neck pain may be attributed to a throat infection, while fever, weakness, increased ESR and CRP may be considered as related to COVID-19 [4]. In Patient 1, SAT diagnosis was delayed despite the obvious symptoms which even preceded COVID-19 manifestation. The problem of delayed SAT diagnosis, especially in patients with preceding symptomatic infection, and of antibiotic abuse in such cases was analyzed by our research team directly before the beginning of the COVID-19 pandemic [30]. We proposed a diagnostic algorithm which included widely available pre-specialist diagnostic tools, starting with neck palpation in all patients with neck pain. In the cases with palpable painful and/or hard thyroid swelling/tumor, ERS and neck US should be performed. Proceeding according to this algorithm can prevent the misuse of numerous antibiotics in patients with SAT, and will help to counteract bacterial antibiotic resistance [30]. In the COVID-19 pandemic, such management has appeared to be even more important, especially in patients with the simultaneous presence of COVID-19 and neck pain.

The presented analysis of the possible correlation between HLA and clinical course of SAT induced by COVID-19, especially with regard to the possible significance of HLA-B*35 homozygosity in the atypically short time lag between the diseases sheds new light on this recently observed phenomenon, and can facilitate management of such patients.

## 5. Conclusions

The unusual phenomenon of the simultaneous occurrence of COVID-19 and SAT triggered by SARS-CoV-2 infection as well as the observed very short time lag between the diseases can be HLA-dependent. The homozygosity at HLA-B*35 can possibly play a role in such an early development of SAT symptoms. Additionally, the clinical course of SAT triggered by COVID-19 can be HLA-related, not only in regard to the previously described risk of recurrence, but also to a variety of other aspects, including severity of thyrotoxicosis. Further research is necessary to confirm these findings.

## Figures and Tables

**Table 1:** Patients’ laboratory tests and clinical findings at the time of SAT diagnosis.

Analyzed Parameter (Reference Range)	Case 1	Case 2	Case 3	Case 4
TSH (0.27–4.2 mIU/L)	1.53	<0.005	0.01	0.07
FT3 (2–4.4 pg/mL)	3.75	21.6	5.13	5.24
FT4 (0.93–1.7 ng/dL)	0.96	>7.7	2.39	2.34
aTPO (<34 IU/mL)	8.6	10.2	10.4	9.4
aTg (<115 IU/mL)	21.6	13.3	512	25.0
TRAb (<1.7 IU/L)	1.58	<0.8	0.8	0.94
ESR (<10 mm/h))	52	59	140	117
CRP (<1 mg/dL)	9.0	60	4.98	4.71
Neck pain	yes	yes	yes	yes
Fever	yes	yes	yes	yes
Sonographic pattern	Several hypoechoic areas in the right thyroid lobe	Several hypoechoic areas in the right thyroid lobe	Several hypoechoic areas in both lobes	Several hypoechoic areas in both lobes
Time lag from COVID-19	Simultanously–SAT was the first symptom of COVID-19	4 weeks	5 weeks	4 months
Remarks	Lab parameters measured after initiation of dexamethasone treatment started due to COVID-19, earlier results not available	Severe clinical symptoms of SAT	Recurrence after SARS-CoV-2 indection, despite prednisolone treatment during the course of COVID-19	

Abbreviations: aTg, thyroglobulin antibodies; aTPO, thyroid peroxidase antibodies; CRP, C-reactive protein; ESR, erythrocyte sedimentation rate; FT3, free triiodothyronine; FT4, free thyroxine; NA, not available; TRAb, thyrotropin receptor antibodies; TSH, thyrotropin.

**Table 2:** HLA genotyping results in the presented patients.

Case No	Gender	HLA- B*35:01 ^1^	HLA- B*35:03 ^1^	HLA- B*18:01 ^1^	HLA- C*04:01 ^1^	HLA- DRB1*01:01 ^1^	HLA- DRB1*03:01 ^2^
1	M	Yes	Yes	No	Yes	Yes	No
2	F	No	Yes	No	No	No	Yes
3	F	Yes	No	Yes	No	No	No
4	F	No	Yes	No	Yes	Yes	No

^1^ SAT risk allele; ^2^ GD risk allele; M, male; F, female.

## Data Availability

Not applicable.

## Abbreviations

aTg	thyroglobulin antibodies
aTPO	thyroid peroxidase antibodies
CRP	C reactive protein
ECLIA	electrochemiluminescence immunoassay
ESR	erythrocyte sedimentation rate
FNAB	fine needle aspiration biopsy
FT3	free triiodothyronine
FT4	free thyroxine
GD	Graves’ disease
HLA	human leukocyte antigens
PASC	post-acute sequalae of SARS-CoV-2 infection
SAT	subacute thyroiditis
TRAb	thyrotropin receptor antibodies
TSH	thyrotropin
US	ultrasound

## References

[1] Stasiak M., Michalak R., Stasiak B., Lewiński A. (2019). Clinical characteristics of subacute thyroiditis is different than it used to be–current state based on 15 years own material. Neuro Endocrinol. Lett..

[2] Stasiak M., Tymoniuk B., Michalak R., Stasiak B., Kowalski M.L., Lewiński A. (2020). Subacute Thyroiditis is Associated with HLA-B*18:01, -DRB1*01 and -C*04:01-The Significance of the New Molecular Background. J. Clin. Med..

[3] Dalugama C. (2018). Asymptomatic thyroiditis presenting as pyrexia of unknown origin: A case report. J. Med. Case Rep..

[4] Stasiak M., Lewiński A. (2021). New aspects in the pathogenesis and management of subacute thyroiditis. Rev. Endocr. Metab. Disord..

[5] Desailloud R., Hober D., Virol J. (2009). Viruses and thyroiditis: An update. Virol. J..

[6] Mangaraj S. (2021). Subacute thyroiditis complicating dengue fever–Case report and brief review of literature. Trop Doc..

[7] Poma A.M., Bonuccelli D., Giannini R., Macerola E., Vignali P., Ugolini C., Torregrossa L., Proietti A., Pistello M., Basolo A. (2021). COVID-19 autopsy cases: Detection of virus in endocrine tissues. J. Endocrinol. Investig..

[8] Li M.Y., Li L., Zhang Y., Wang X.S. (2020). Expression of the SARS-CoV-2 cell receptor gene ACE2 in a wide variety of human tissues. Infect. Dis. Poverty.

[9] Scappaticcio L., Pitoia F., Esposito K., Piccardo A., Trimboli P. (2020). Impact of COVID-19 on the thyroid gland: An update. Rev. Endocr. Metab. Disord..

[10] Puelles V.G., Lütgehetmann M., Lindenmeyer M.T., Sperhake J.P., Wong M.N., Allweiss L., Chilla S., Heinemann A., Wanner N., Liu S. (2020). Multiorgan and Renal Tropism of SARS-CoV-2. N. Engl. J. Med..

[11] Chen W., Tian Y., Li Z., Zhu J., Wei T., Lei J. (2021). Potential Interaction Between SARS-CoV-2 and Thyroid: A Review. J. Endocrinol..

[12] Muller I., Cannavaro D., Dazzi D., Covelli D., Mantovani G., Muscatello A., Ferrante E., Orsi E., Resi V., Longari V. (2020). SARS-CoV-2-related atypical thyroiditis. Lancet Diabetes Endocrinol..

[13] Brancatella A., Ricci D., Viola N., Sgrò D., Santini F., Latrofa F. (2020). Subacute Thyroiditis After Sars-CoV-2 Infection. J. Clin. Endocrinol. Metab..

[14] Campos-Barrera E., Alvarez-Cisneros T., Davalos-Fuentes M. (2020). Subacute Thyroiditis Associated with COVID-19. Case Rep. Endocrinol..

[15] Lania A., Sandri M.T., Cellini M., Mirani M., Lavezzi E., Mazziotti G. (2020). Thyrotoxicosis in patients with COVID-19: The THYRCOV study. Eur. J. Endocrinol..

[16] Ippolito S., Dentali F., Tanda M.L. (2020). SARS-CoV-2: A potential trigger for subacute thyroiditis? Insights from a case report. J. Endocrinol. Invesigt..

[17] Asfuroglu Kalkan E., Ates I. (2020). A case of subacute thyroiditis associated with COVID-19 infection. J. Endocrinol. Investig..

[18] San Juan M.D.J., Florencio M.Q.V., Joven M.H. (2020). Subacute thyroiditis in a patient with Coronavirus disease 2019. AACE Clin. Case Rep..

[19] Ruggeri R.M., Campennì A., Siracusa M., Frazzetto G., Gullo D. (2021). Subacute thyroiditis in a patient infected with SARS-CoV-2: An endocrine complication linked to the COVID-19 pandemic. Hormones.

[20] Brancatella A., Ricci D., Cappellani D., Viola N., Sgrò D., Santini F., Latrofa F. (2020). Is Subacute Thyroiditis an Underestimated Manifestation of SARS-CoV-2 Infection? Insights from a Case Series. J. Clin. Endocrinol. Metab..

[21] Mattar S.A.M., Koh S.J.Q., Rama Chandran S., Cherng B.P.Z. (2020). Subacute thyroiditis associated with COVID-19. BMJ Case Rep..

[22] Trimboli P., Cappelli C., Croce L., Scappaticcio L., Chiovato L., Rotondi M. (2021). COVID-19-Associated Subacute Thyroiditis: Evidence-Based Data from a Systematic Review. Front. Endocrinol..

[23] Brancatella A., Viola N., Rutigliano G., Sgrò D., Santini F., Latrofa F. (2021). Subacute Thyroiditis During the SARS-CoV-2 Pandemic. J. Endocr. Soc..

[24] Patients with COVID-19 May Develop Thyroid Infection. 21 May 2020. https://www.endocrine.org/newsandadvocacy/newsroom/2020/patientswithcovid19maydevelopthyroidinfection.

[25] Stasiak M., Tymoniuk B., Adamczewski Z., Stasiak B., Lewiński A. (2019). Sonographic Pattern of Subacute Thyroiditis Is HLA-Dependent. Front. Endocrinol..

[26] Stasiak M., Tymoniuk B., Stasiak B., Lewiński A. (2019). The Risk of Recurrence of Subacute Thyroiditis Is HLA-Dependent. Int. J. Mol. Sci..

[27] Stasiak M., Lewiński A. (2020). Strong Correlation between HLA and Clinical Course of Subacute Thyroiditis-A Report of the Three Siblings. Genes.

[28] Desimmie B.A., Raru Y.Y., Awadh H.M., He P., Teka S., Willenburg K.S. (2021). Insights into SARS-CoV-2 Persistence and Its Relevance. Viruses.

[29] Samuels M.H. (2012). Subacute, silent, and postpartum thyroiditis. Med. Clin. N. Am..

[30] Stasiak M., Michalak R., Stasiak B., Lewiński A. (2020). Time-Lag Between Symptom Onset and Diagnosis of Subacute Thyroiditis–How to Avoid the Delay of Diagnosis and Unnecessary Overuse of Antibiotics. Horm. Metab. Res..

